# Chronic Low Back Pain: History, Symptoms, Pain Mechanisms, and Treatment

**DOI:** 10.3390/life14070812

**Published:** 2024-06-27

**Authors:** Tyler Farley, Jesse Stokke, Kush Goyal, Russell DeMicco

**Affiliations:** Center for Spine Health, Neurological Institute, Cleveland Clinic, 9500 Euclid Ave, Cleveland, OH 44195, USA; stokkej@ccf.org (J.S.); goyalk@ccf.org (K.G.); demiccr@ccf.org (R.D.)

**Keywords:** chronic low back pain, lumbar facet arthropathy, discogenic low back pain, vertebrogenic low back pain, sacroiliac joint, axial low back pain, mechanical low back pain, lumbar pain, lumbosacral degenerative disease

## Abstract

Chronic low back pain (cLBP) is the most frequently reported cause of years lived with disability. Identifying the anatomical structures or dysfunction contributing to patients’ symptoms is critical to guiding treatment. The etiology of back pain and differential diagnosis is often broad, ranging from non-degenerative cLBP (trauma, tumor, inflammation, infection, etc.) to degenerative (also described as nonspecific) cLBP. After eliminating suspicion for more insidious causes of cLBP, a thorough investigation can be conducted in an attempt to identify a source of degenerative cLBP. Degenerative cLBP can originate from many sources, and a detailed understanding of the structures potentially involved is invaluable for an accurate diagnosis. This review article aims to provide a broad overview of the utility of clinical history, physical exam findings, imaging findings, and diagnostic procedures in identifying the cause of patients’ cLBP. We provide a framework to help guide clinicians by dividing the structures into groups as follows: anterior vertebral column, posterior vertebral column, and extra-vertebral pain. For each condition listed, we touch on the treatment options that can be considered.

## 1. Introduction

A systematic analysis of the global burden of disease determined that chronic low back pain (cLBP) has been a leading cause among 354 diseases for years lived with disability from 1990 to 2017 [1,2]. The advances in management with the greatest potential are likely ones that align practice with evidence and promote activity and function with continued functional participation in life and work [3]. This highlights the need for a detailed understanding of cLBP and how to address the treatment of these patients.

A variety of definitions exist for the definition of cLBP, ranging from inveterate pain in the lumbar region for longer than 3 months (the French agency for healthcare evaluations, Agence NAtionale d’Évaluation en Santé, ANAES), to low back pain with a duration of at least 6 months (the American Society of Interventional Pain Physicians, ASIPP) [4,5]. The term “nonspecific low back pain” was coined based on the foundation of a study from 1966, which concluded that the source of cLBP could not be identified in 79% of males and 89% of females in a general practice setting [6]. In the ensuing years, this conclusion has been repeated despite the advances in imaging, diagnostic modalities, and specialized care in the field of spine medicine. Although physicians’ ability to accurately identify the cause of patients’ back pain has undoubtedly improved, diagnosis often remains challenging.

Low back pain can be characterized as neuropathic, nociceptive, or nociplastic (pain that arises from altered nociception despite no clear evidence of nociceptive or neuropathic pain) [7]. A new classification of cLBP establishes three categories of pain based on presumptive mechanisms and whether the pain is related to degenerative changes. Non-degenerative cLBP describes pain caused by pathology such as trauma, spondylolysis, tumors, infections, or inflammatory processes. As such, cLBP can represent a serious medical pathology that requires an appropriate diagnostic workup or specialist referral [1]. The cLBP of an unknown mechanism is pain that seems to bear no relation to anatomical abnormalities, for which the Fear Avoidance Beliefs Questionnaire and other similar scales may be useful. Finally, degenerative cLBP has replaced the term “nonspecific low back pain” to describe pain attributable to degenerative alterations in discs, facet joints, and/or ligaments, with or without regional and/or global alterations in spinal alignment [8].

History-taking is an important aspect to help narrow the differential diagnosis, aid in focusing physical exams, and can be used to individualize treatment plans. The first part of this section will focus on gathering an appropriate medical history of the patient’s pain, discussing “Red” and “Yellow” flags regarding cLBP, as well as briefly discussing other pertinent histories that may influence the development of cLBP, treatment decisions, prognosis for recovery, and exploring etiologies that may mimic cLBP. Body systems that are discussed are not meant to be viewed as all-inclusive; however, a focus on the relevant aspects of medical history-taking for cLBP patients is needed.

## 2. Evaluation of Pain

A thorough history of pain aims to gather features of cLBP that can be used to focus pertinent questions, the physical exam, and explore other co-morbid conditions that may influence treatment.

Onset of pain: How long has the patient experienced low back pain? Onset will often include documenting a specific inciting event if one occurred, such as pain that started after a motor vehicle accident or a fall at work versus pain that started without trauma or a specific injury/event.

Progression: How pain has progressed should be documented. Pain that has been present for years but specifically worsened over the past month may change diagnostic or treatment considerations.

Location: The location of pain should include the side (right, left, or bilateral), in the midline, paraspinals, buttocks, or radiation down the posterior/anterior thighs. At times, patients will describe diffuse, widespread spinal pain. The next chapter will dive deeper into the physical exam and diagnostic confidence of physical exam tests that a provider may use to further investigate pain that is suggested by location (i.e., paraspinal for facets, or buttock for SI joint or hip pathology) and other history-taking aspects.

Description/characteristic: Descriptions often vary between dull, achy, sharp, stabbing, stiff/tight, crampy, and/or burning. Specific descriptions that may be helpful and can elucidate potential systemic involvement, such as morning stiffness >30 min, may raise suspicion for inflammatory back pain vs. degenerative cLBP.

Intensity/Severity: Patients are asked to describe the pain on a number rating from zero to ten out of ten (0–10/10) (NRS) or a visual Analog scale (VAS). Ex: 4/10 pain with rest that increases to 7/10 pain with lumbar extension.

Aggravating and alleviating factors: Aggravating factors include what provokes or makes the patients’ back pain worse. Focusing on specific positions (sitting vs. standing), movements (flexion, extension, laying down, walking, etc.), or time of day (morning vs. evening) when pain is more intense. Alleviating factors are the opposite; e.g., what position reduces the pain. Often patients will describe a treatment option in response to the questions, e.g., whether pain is better with ice or after medication.

Radiation of symptoms: Have patients describe radiation outside of the lumbar spine and past the gluteal folds. Pain that has a dermatomal or radicular pattern down the entire lower extremity often raises suspicion for involvement of neural components, which falls outside the discussion of degenerative cLBP.

Other associated symptoms: Symptoms that may raise suspicion for other processes distinct from degenerative changes in the spine include weakness, numbness/tingling, fevers/chills, weight loss, pain that wakes them up at night, changes in bowel/bladder control, balance issues, tremors, fasciculations, or abdominal pain, just to name a few.

Other important aspects of chronic pain that clinicians should inquire about and document are current and past responses to medical treatments. Physical therapy, medications (opioids, muscle relaxants, NSAIDs, etc.), injections along with response, and past surgical history of the back, as well as a complete surgical history, should be gathered. Other treatment modalities, including acupuncture, tai chi, yoga, pilates, manipulation, massage therapy, electrical stimulation, traction, heat/ice packs, topicals, or patches, are also treatment options with varying degrees of recommendations and levels of evidence. If yoga specifically was helpful for a patient’s myofascial pain for 2 years, it would be worth attempting to return to yoga.

## 3. Red Flags

The term “red flags” is used to describe history, signs, and symptoms that might suggest secondary causes of a more serious underlying pathology for low back pain. First described in the 1980s [9], there are now numerous terms, guidelines, and lists that lack a census of what might be considered a red flag. A comprehensive review published in 2016 in the European Spine Journal identified 21 guidelines by various countries and associations and included 16 in their review to compare recommendations [10]. The most commonly referred pathologies to which red flags may be indicative included malignancy, fracture, infection, and cauda equina syndrome (CES), with other mentions including ankylosing spondylitis, aneurysms, and myelopathy [10]. It has been noted that serious red flag conditions presenting as low back pain are rare [11]. Clinicians should be aware of patient history and inquire about new or progressive symptoms on subsequent visits. The four most common pathologies mentioned in the review red flag guidelines include the following:

Malignancy: Fourteen red flags were identified related to malignancy, with a history of cancer being found in all guidelines, and unintentional weight loss was the second most common. Other mentions included pain at rest or pain at night, elevated ESR, reduced appetite, rapid fatigue, fever, paraparesis, progressive symptoms, and strong clinical suspicion [10].

Fracture: Eleven different red flags noted for indication of a potential fracture were found. Major or significant trauma is the most common, followed by the use of steroids or immunosuppression, old age (often between 50 and 70 years old), female gender, previous fracture, low body weight, increased thoracic kyphosis, structural deformity, or minor trauma [10].

Infection: The most common recommendations related to infection were fever, use of corticosteroids, immunosuppression, intravenous (IV) drug use, pain at night or at rest, and bone tenderness of spinous processes [10].

Cauda Equina Syndrome (CES): Overall, nine different red flags were described for suspicion of CES, with new-onset saddle anesthesia (perineal numbness) and bladder dysfunction being the two most common [10].

## 4. Yellow Flags

The term “yellow flags” was first identified in 1997 by Kendall et al. [12], after a collaborative project in New Zealand by the National Health Committee and the Accident Rehabilitation Compensation Insurance Corporation (ACC) that described psychosocial risk factors for long-term disability and work loss related to low back pain [13]. Since then, there has been an increased focus on the connection between psychosocial and workplace factors as they relate to cLBP. The North American Spine Society’s (NASS) evidence-based review of cLBP provided a Grade A recommendation (highest level of evidence) regarding the evaluation of a patient’s psychosocial and workplace factors, citing their contribution to the development of disabilities related to cLBP [14]. The European Guidelines for Chronic Low Back Pain describe yellow flags as psychosocial risk factors, including (1) inappropriate attitudes and beliefs about back pain, such that pain may be harmful and potentially disabling, or having elevated expectations that passive treatments rather than active treatments will be helpful. (2) Inappropriate pain behaviors such as fear avoidance, and reduced levels of activity related to fear of worsening pain. (3) Compensation or work-related issues. (4) Emotional problems including depression, anxiety, stress, and withdrawal from social interaction. Identification of yellow flags, during medical history or screening questionnaires should prompt providers for appropriate cognitive and behavioral management as part of the ongoing treatment plan [15].

### Psychosocial

Pain is a personal experience built on an individual’s life experiences. The revised International Association for the Study of Pain’s (IASP) definition of pain is “an unpleasant sensory or emotional experience associated with actual or potential tissue damage” [16]. With the idea of potential tissue damage being a component of an individual’s experience of pain, the fear avoidance model is one of the most influential models used to explain how psychological factors affect this experience and play into the development of chronic pain, inactivity, depression, and disability [17].

Although depression and anxiety have not been identified as a specific cause of back pain, substantial data show that depression and anxiety rates are found to be higher in individuals with cLBP compared to the general population [18,19,20,21,22,23,24,25]. CLBP can create a difficult cycle to break when a reduction in movement leads to reduced pain, further leading to maladaptive beliefs regarding pain such as catastrophizing, kinesiophobia, or fear avoidance (normal movements will cause pain), and further psychological distress, which has been shown to be bi-directional and lead to a more severe experience of pain [24,25]. These contributions are part of what make cLBP one of the more challenging conditions for recovery. To help identify co-morbid psychosocial factors, clinicians can use multiple screening questions such as the Patient Health Questionaire-9 (PHQ-9), General Anxiety Disorder-7 (GAD-7), or Fear Avoidance Belief Questionnaire. Identification of these disorders is a key component of history-taking and can offer options to individualize treatment goals and protocols for cLBP patients.

Other psychological backgrounds that can be valuable to obtain during history include PTSD or trauma history (sexual, physical, or emotional abuse), which has been found to be up to 4× more common in chronic pain patients compared to the general population [26]. There is a large body of evidence that suggests that occupational factors have a substantial impact on a patient’s ability to develop chronic pain [27]. This includes those in blue-collar jobs, heavy labor jobs, workers with low job satisfaction, those who are new at their job, and workers who are not well rated by their superiors [27]. Although not as robust data is currently present, studies out of Germany have shown financial strain and debt to be a risk factor for back pain [28].

## 5. Lifestyle

### 5.1. Function and Disability

The Global Burden of Disease, a systematic analysis of 354 diseases across 195 countries in 1990 and 2017, found low back pain to be the leading cause of years lived with disability (YLD) [2]. Globally, YLDs of LBP increased 52.7% from 1990 to 2017 [2]. The burden of low back pain globally is undeniable, and its effect on a patient’s mood, beliefs, social interactions, and functional or non-functional status is inherently tied together. Thus, an overall goal for the treatment of cLBP should be to increase a patient’s daily function and limit disability. To assist in the subjective evaluation of patients’ function and disability, there have been numerous tools and questionnaires developed, referred to as Patient-Reported Outcome Measures (PROMs). Regarding back pain and function, commonly used PROMs include the following: Pain Disability Assessment Scale (PDAS), Patient Reported Outcome Measurement Information System (PROMIS), Oswestry Disability Index (ODI), Oswestry Low Back Pain Disability Questionnaire (OSW), Roland Morris Pain Scale, Short Form-36 (SF-36), and the Quebec Back Pain Disability Scale (QUE). Originally developed to compare the effectiveness of treatment in research and clinical trials [29], PROMs have been increasingly used to monitor patient response to treatment. Overall, to our knowledge, no study has compared the validity or reliability of all developed measurement tools. Clinicians should use the assessment tool they find most relevant and appropriate [30]. One goal of assessment tools should be to help clinicians gain insight into the effect that cLBP has on an individual’s life.

The physical activity level of patients should be identified and discussed as part of their medical history. Although some studies suggest no conclusive evidence that adults with cLBP are less active than healthy individuals [31,32], most of the literature supports the conclusion that a sedentary lifestyle and decreased physical activity are found in patients with cLBP [33,34,35,36,37]. When further looking at subgroups of cLBP and activity distribution, it has been seen that elderly patients (>65) with cLBP have decreased physical activity compared to controls [38], cLBP is associated with significantly less time walking, take a fewer number of steps per day [39], spend less time laying down during the day [40], and even one of the studies that found no conclusive evidence for the overall activity difference noted cLBP are less active in the morning compared to pain-free individuals [32], suggesting a difference in the distribution of activity.

### 5.2. Sleep

Impaired sleep is known to have a correlation with cLBP [41,42,43], with >50% of patients with cLBP complaining of sleep disturbance. Not only sleep impairment in general, but specifically, there is a direct correlation with pain intensity and function related to cLBP [44]. Impairments can include reduced sleep quality, difficulty falling asleep, waking up after sleep, and feeling unrefreshed from sleep. Treatment focusing on sleep hygiene, prevention of, and reduction in sleep disturbances has been suggested to correlate to a higher probability of recovery and improve the long-term prognosis of cLBP [45].

### 5.3. Smoking

A history of smoking is important to assessing the overall health of patients and should be a standard component of medical history for all evaluations. Smoking is a known correlation to adults who experience cLBP [33,46]. Recent studies aimed at smoking cessation have demonstrated pain status as a predictor of cessation outcomes [47]. This highlights unique barriers for those who experience pain and smoke and the utility of addressing pain among smokers who are planning or anticipating an upcoming attempt at cessation [47]. It has also been established that smoking increases overall rates of malignancy [48]. Additionally, nicotine use is known to affect healing and thus can affect recovery from a spine-related injury [49,50]. Furthermore, the cost of smoking may negatively affect a patient’s finances and ability to focus resources on health care treatment, such as physical therapy or medication.

## 6. Co-Morbid Conditions

### 6.1. Cardiovascular

Cardiovascular disease (CVD) is the leading cause of mortality worldwide [51,52,53,54]. Given the vast implications of CVD on a patient’s overall health, this section will briefly touch on certain aspects of CVD related to cLBP and should not be seen as a complete guide as it relates to cardiovascular implications. The relationship with cLBP has been seen across numerous studies, showing higher incidence not only with cardiovascular disease [55,56,57], heart failure [58,59], and hypertension [33,60], but also higher rates with known cardiovascular risk factors such as high cholesterol, high LDL, high triglycerides [61,62], obesity [63,64], smoking [33,46], and diabetes [33,65,66]. The data also point toward the fact that the more severe the back pain, the more likely individuals are to have a myocardial infarction [56,67]. The idea that pain may be more common in patients with cardiovascular disease is multifactorial. Heart failure patients with pain tend to be older, and in particular, those >85 years of age tend to experience more frequent chronic pain [68,69]. Additionally, HF is correlated with high rates of depression [59,70,71], which has already been discussed as being correlated with cLBP. HF patients tend to exercise less and have decreased physical function [59,72,73,74], and even CVD and cLBP have been hypothesized via atherosclerotic processes of the abdominal aorta [75].

Knowing a patient’s cardiovascular history can stop putting patients at greater risk when considering their medication treatment options. Non-aspirin non-steroidal anti-inflammatory medications (NSAIDs) are a frequently used class of medications for pain. Understanding that there are increased cardiovascular events [76,77,78] with these medications may reduce future adverse events. Additionally, patients who have had prior MI or CVAs may already be prescribed blood-thinning medications, and NSAIDs, again, are recommended to be avoided [79]. Benefits have been seen in treating HF patients and co-morbid depression with cognitive behavioral therapy (CBT) combined with exercise training vs. CBT alone and exercise training alone [80]. Thus, given the multifactorial nature of cLBP in the setting of CVD, an individualized treatment plan incorporating improved physical activity, endurance, and proper treatment of co-morbid depression is an avenue to improve quality of life. The treatment of cLBP needs to be weighed alongside the patient’s current cardiac status. For example, it may be best to recommend cardiac rehabilitation before starting low-back pain-targeted physical therapy. Finally, in patients suffering from active cardiac issues, PCP, and/or cardiac referral should be made immediately.

### 6.2. Rheumatologic

#### 6.2.1. Rheumatoid Arthritis (RA)

RA is one of the most common inflammatory diseases worldwide. The frequency of cLBP in RA patients has been shown to be much higher than in the general population, ranging between 33 and 65% [81,82,83]. RA causes erosive changes to joints throughout the body, and these changes can affect the spine, including endplate and facet erosions [84]. These changes have been hypothesized to be a result of pathologic changes to the spine due to moderate-to-high disease activity, as opposed to a degenerative process [84,85], and can lead to more severe cLBP among RA patients [85]. Other factors correlated to more severe cLBP in this population include a higher BMI, less frequent use of methotrexate, and smoking [84,85]. In addition, upper cervical spine pathology and corresponding symptoms, such as myelopathy, should be considered in patients with RA.

#### 6.2.2. Axial Spondyloarthritis (axSpA)

AxSpA is a term used to describe inflammatory conditions of the spine and sacroiliac joints. AxSpA includes ankylosing spondylitis (AS), now sometimes referred to as radiographic and non-radiographic axial spondyloarthritis (r-axSpA and nr-axSpA), spondyloarthritis associated with inflammatory bowel disease, and undifferentiated spondyloarthritis [86,87]. The understanding of these conditions has grown over the last two decades. As the topic of this chapter’s focus is the medical history of degenerative cLBP, only pertinent aspects of inflammatory axSpA will be touched on, specifically medical presentation and diagnosis.

Diagnosis of AS has been difficult for the general medical community, due to the heterogeneity and multifactorial nature of cLBP. The data suggest median times from first presentation of symptoms to diagnosis ranging from 3.8 to 8.3 years, and it is often quoted as taking longer than 10 years to diagnose [87]. Patients often present with descriptions of inflammatory back pain, that is, pain and stiffness that is worse in the morning, stiffness lasting >30 min, and stiffness that is improved with exercise and NSAIDs [87,88]. AS is most commonly present in males, with a male-to-female ratio ranging from 2:1 to 5:1, and the peak age of onset is 15–35 years old, often before the age of 40 [87,88]. Other parts of a patient’s history that should raise a clinician’s suspicion for axSpA are inflammation of the tendon insertion to bone (enthesitis) [87]. These frequently occur at the Achilles but can also occur at the knees, shoulders, hips, and chest wall, and they are seen in 30–50% of patients with axSpA [87]. Dactylitis (swelling of the entire finger or toe) and uveitis (eye inflammation that can cause pain, swelling, and blurred vision) are seen in 6% and 10%, respectively, of axSpA [87]. Skin changes should also be taken note of, as psoriasis (dry, itchy, and scaly patches) is seen in 10% of axSpA. Inflammatory bowel disease is also seen in about 5–10% of axSpA, and evidence of microscopic colitis (noted on colon biopsy) is more frequently seen in about 30–60% [87].

Clinicians should be aware of the diagnostic criteria for these conditions. For AS, the modified New York Classification Criteria was described in 1984. However, for a definitive diagnosis, patients would need to have x-ray images showing radiographic changes seen in the SI joints along with at least one clinical sign, and only possible AS could be defined clinically [88]. Evidence that radiographic changes can take 6–10 years after symptom presentation [89] and the advancement of MRI prompted the publication of the Assessment of SpondyloArthritis International Society (ASAS) in 2009. For these patients who have >3 months of back pain and onset under the age of 45, they either need an identification of sacroiliitis on imaging (x-ray or MRI) plus 1 SpA clinical feature or positive HLA-B27 and 2 Spa clinical features for a diagnosis of axSpA [90,91].

### 6.3. Diabetes

The prevalence of type 2 diabetes mellitus (T2DM) has increased over the last few decades, and it affects an estimated 6% of the world’s population [92]. With cLBP and T2DM being so prevalent worldwide, clinicians will often see patients who are affected by both conditions. Some studies have found that the likelihood of cLBP is 3x greater in patients with T2DM (57). Diabetic glycosylation and microangiopathy can also affect both the tissue matrix and nutrition of the spine and soft tissues, leading to more prominent degenerative changes [93,94,95], as a longer duration and poor control of T2DM have been found to correlate with more severe lumbar degeneration [94]. Promoting tight glucose and physical activity has been shown to prevent or delay the advancement of lumbar disc degeneration, decrease the incidence of low back pain, and subsequently reduce disability [94,96].

### 6.4. Malignancy

A personal history of malignancy should be noted for all patients who present with cLBP, as spinal metastases are estimated to affect anywhere between 15 and 70% of cancer patients [97,98,99]. Although all primary malignancies can spread to the spine, the most common malignancies include breast (21%), lung (19%), prostate (7.5%), renal (5%), gastrointestinal (GI) (4.5%), and thyroid (2.5%) [99]. A study by Shakil et al., which identified 37,375 patients with spinal metastasis between 2009 and 2017, found that lung cancer had the highest annual incidence rate (7.8%) after just one year of primary diagnosis, increasing to 10.3% after 10 years, and lung cancer had the greatest risk of metastasis of all cancers after diagnosis [100]. The study revealed that all of the cancers identified (lung, breast, prostate, GI, melanoma, and urologic) increased the incidence of spinal metastasis from 1 year, 5 years, or 10 years after primary diagnosis [101], showing the years from diagnosis to be a risk factor. The highest locations of metastatic lesions in the spine are thoracic (60–80%), lumbar (15–30%), and cervical (<10%) [102,103]. Pain is the hallmark presenting sign of spinal metastasis. Other symptoms can be weakness (35–75%), sensory disturbances, and weight loss [100,101,102,103]. Pain can be described as aching and deep, worsened pain at night, and it can wake patients from sleep [100,103].

### 6.5. Gynecologic

For female patients of childbearing age who present with cLBP, their obstetric and gynecologic history should be considered. Endometriosis is one of the most widespread gynecologic disorders and causes of pelvic pain. It peaks between the ages of 25 and 29, affecting 10–15% of fertile women and 21–40% of infertile women [104]. Suspicion for endometriosis should be raised when pain is in the pelvic region, lower abdomen, lumbar spine, buttocks, or thighs, along with period-related pain (dysmenorrhea), cyclical abdominal symptoms such as painful bowel movements during periods, pain during or after intercourse (dyspareunia), and painful cyclical urinary symptoms, with or without blood in the urine. Ovulation pain, also known as Mittelschmerz, is defined as preovulatory lower abdominal pain that occurs mid-cycle, typically days 7 through 24. Mittelschmerz is estimated to affect up to 40% of women of reproductive age and should also be considered in reproductive-aged women who describe cyclical back pain and abdominal pain related to ovulation [105].

### 6.6. Nephrology and Urologic

Kidney stones (nephrolithasis) or urethral stones (urolithiasis) are a known process that can refer to pain in the lower back or the inferior costovertebral region. Patients may report a known history of kidney stones; however, without a previous diagnosis, this may present the work-up of back pain that is often described as waves of sudden onset and severe pain of the flank or inferior posterior costovertebral region that may radiate to the inferior abdomen, groin, or genitals. History may also reveal nausea, vomiting, pain with urination (dysuria), urinary frequency, urgency, or episodes of blood in the urine (hematuria) [106].

Chronic prostatitis (bacterial or abacterial) occurs in 4.5–9% of males, and incidences increase with age [107]. Pain and symptoms are more common in the urogenital and anorectal regions, but they can also include the suprapubic, lower abdominal, and lumbar regions, which can pose challenges for clinicians and often lead to misdiagnoses. Bowel and bladder function questionnaires may reveal a history of increased urinary frequency, especially at night (nocturia), urgency, a weak urinary stream, or rectal pain during or after defecation [107].

When treating cLBP, chronic kidney disease (CKD), end-stage renal disease (ESRD), and post-kidney transplant are important comorbidities that general practitioners and specialists should be mindful of. Medication regimens may need to be adjusted, avoided, or closely monitored for side effects. Common medication classes used for management of cLBP that have modified clearance, effects on kidney function, or that should be avoided depending on the degree of kidney function include the following: NSAIDs, opioids, tramadol, pregabalin, gabapentin, SSRIs, SNRIs, and TCAs [108,109]. Clinicians should be aware of potential interactions and monitor both kidney function, side effects, and interactions with other medications when treating cLBP with comorbid kidney disease.

Although advances in diagnostic modalities, including imaging, have aided the ability to identify the causes of cLBP, the clinical exam remains invaluable. As the burden of cLBP is growing alongside aging populations and because these population shifts are more rapid in low-income and middle-income countries with limited resources to diagnose and address the problem, the clinical exam and history-taking remain invaluable [110]. We will also discuss typical symptoms that can be helpful when taking a history and their potential clinical implications. For each cause of cLBP, we will also briefly touch on diagnostic and therapeutic treatment options. As the burden of cLBP falls largely on the adult population, this review article will focus on such patients.

When evaluating patients presenting with cLBP, it is reasonable to divide the etiology into pain arising either from the vertebral column or pain of adjacent axial structures. The elements of the lumbar spine include soft tissues, bony structures and joints (vertebrae, spinous processes, zygapophyseal, and sacroiliac joints), intervertebral discs, and neurovascular structures, all of which are common pain generators. 

## 7. Vertebral Column Pain

Regarding the vertebral column, the cause of pain may be further divided into being attributed to any of the following three regions: the anterior, neural, or posterior elements of the spine. As the neural elements can present more predictably as symptoms radiating down the lower extremity and this review is focusing on cLBP, we will refrain from discussing pain of neurogenic claudication or radicular etiology.

### 7.1. Anterior Element Pain

For the purposes of this review, the anterior vertebral column refers to the vertebral bodies and discs. Previously, pain stemming from the discs, or “discogenic pain”, was thought to be the cause of cLBP for 39–42% of patients [111]. It was thought that pathological neurovascular ingrowth penetrated into annular fissures, leading to increased sensitivity and nociception via the sinuvertebral nerve [112]. However, more recent evidence appears to refute the occurrence of such neurovascular ingrowth in many cases [113]. For the histomorphology of human vertebral bodies demonstrated endplate nociceptor densification in areas of damage that were associated with increased disc degeneration. In addition, they found that only 30% of annular tears in degenerated discs had pathologic neural ingrowth, compared with 90% of adjacent endplates, which were twice as densely innervated. Nerve ingrowth into the disc is likely inhibited by physical pressure and proteoglycans and limited to proteoglycan-deficient annular fissures [8].

A branch of the sinuvertebral nerve, the basivertebral nerve, enters the vertebral body posteriorly through a foramen and travels cephalad and caudally to densely innervate the vertebral end plates [114]. As this nerve is unmyelinated and has not shown potential for regeneration, it has become a therapeutic target where radiofrequency ablation of this nerve has shown long-term relief of cLBP, with the results being published for up to 5 years [115].

As a result, the vertebral end plates have been an area of recent interest as a cause of nonspecific low back pain. Changes in the adjacent end plates of degenerative discs were first described radiologically with magnetic resonance imaging (MRI) by Modic in 1988 [116]. These changes were first divided into two types, with Type 1 endplate (or Modic) changes having increased signal on T2-weighted imaging and decreased signal on T1-weighted imaging, and Type 2 changes having increased signal on T1 and increased or isointense signal on T2. Type 3 changes are a result of extensive sclerosis and are represented on an MRI as a decreased signal for both T1 and T2 sequencing. Type 1 endplate changes are thought to be a result of fibrovascular replacement or edematous changes in the subchondral bone, and Type 2 changes are likely a result of fatty replacement.

Approximately 22% (12–37%) of patients with nonspecific cLBP have Modic changes type 1 (MC1) or mixed Modic changes Type 1/Type 2 (MC1/2) [117]. These findings can be seen in both symptomatic and asymptomatic patients [118]. A systematic review and meta-analysis identified no strong association between end plate changes and MRI and cLBP in patients with disability, although there is a need for further research as this study exhibited significant heterogeneity of clinical outcomes and published articles [119]. However, a re-analysis of the literature focusing on a homogenous patient population concluded that the evidence is more clear, suggesting that Modic changes are associated with cLBP and that Type 1 changes may be more painful than Type 2 [120].

Despite this, the intervertebral discs themselves are also associated with pain. Patients with disc degeneration are two to three times more likely than those who do not have a degenerative disc to experience back pain [121]. The discs can cause pain by a number of mechanisms, as follows: neuroinflammation-induced innervation of nociceptive fibers in the disc, neuroinflammation-induced nociception, and pain from annular tears, among others [122].

This makes anterior column pain a diagnostic challenge to distinguish between discogenic or vertebrogenic pain, as there is significant overlap between the etiology and pathogenesis of the condition and both structures are stressed by the same positions. At minimum, however, we can identify factors that would indicate pain arising from the anterior column. To begin with, the pain should be primarily midline, with minimal pain referred to bilaterally. History and physical exam components that are consistent with discogenic or vertebrogenic should theoretically correlate with activities that increase the pressure on these spinal components. In the 1960s, Nachemson et. al. placed a pressure transducer into the intervertebral disc to measure the intradiscal pressure directly. This classic study concluded that the intradiscal pressure increased with bending or sitting, especially with poor posture, in comparison to standing [123]. More recent studies challenge these findings and suggest that sitting may not increase this pressure as much as previously thought. A study that estimated disc heights radiographically suggested that standing may impose higher loads on the discs than sitting [124]. A systematic review and meta-analysis that analyzed in vivo studies evaluating intradiscal pressure while standing or sitting concluded that the sitting posture induced significantly higher intradiscal pressure on the lumbar spine than standing. It also concluded that there was no difference in sitting vs. standing intradiscal pressure in studies published after 1990 or in study subjects who were diagnosed with degenerated discs [125]. Regardless, lifting (especially with objects further from the body) and bending (forward flexion) are well-established mechanisms of increased intradiscal pressure that may be able to fairly reliably indicate patients who have discogenic or vertebrogenic pain generators, particularly in the appropriate clinical context combined with imaging findings [126,127]. Nevertheless, a provocative discogram, where contrast or another injectate is instilled into the disc utilizing fluoroscopy or computed tomography for guidance, remains the gold standard for the diagnosis of a painful disc [128]. There is a paucity of high-quality evidence for procedures for discogenic pain (as discussed in the next paragraph), and studies have raised concern for further degeneration and problems with the intervertebral disc after discogram. Therefore, this diagnostic procedure’s clinical utility may be limited [129].

Various interventions have historically been performed for discogenic pain. Chemonucleolysis is an intervention where the disc is injected with substances that cleave water molecules from proteoglycans (and other substances) in the nucleus pulposus to decrease the size of disc protrusions or herniations. Since this is primarily performed for radicular pain, we will refrain from discussing it further. A 2010 study showed promise for injecting methylene blue into the disc due to its anti-inflammatory effects from inhibiting the production of nitric oxide [130]. Unfortunately, the results were not supported in subsequent larger-scale studies [131]. In the 1990s and 2000s, we saw a surge in minimally invasive techniques to treat discogenic low back pain, including intradiscal electrothermal therapy (IDET) or percutaneous intradiscal radiofrequency thermocoagulation, essentially with the goal of ablating painful nerve fibers at annular tears. Randomized controlled trials concluded that there is insufficient evidence to recommend these procedures for the treatment of discogenic low back pain [132]. There is a substantial amount of research currently looking into the utility of orthogiologic restorative or regenerative treatments for the degenerated intervertebral disc. Such treatments include platelet-rich plasma, various stem cell products, and hydrogel injections. As the disc’s pH is too low and the oxygen and nutrition diffusion into the inner annulus is low in the degenerated disc, the viability of cells added to the nucleus pulposus is low [133]. For this reason, various cell delivery mechanisms are being developed with matrices and other vehicles to help restore the structure and enable the survival of the injected cells [134]. At present, the evidence for the use of intradiscal biologic treatments is very low, leaving this an area that should receive further research, with exciting potential for overcoming its obstacles [135].

### 7.2. Posterior Element Pain

#### 7.2.1. Facet Joint Pain

**Figure 1:**
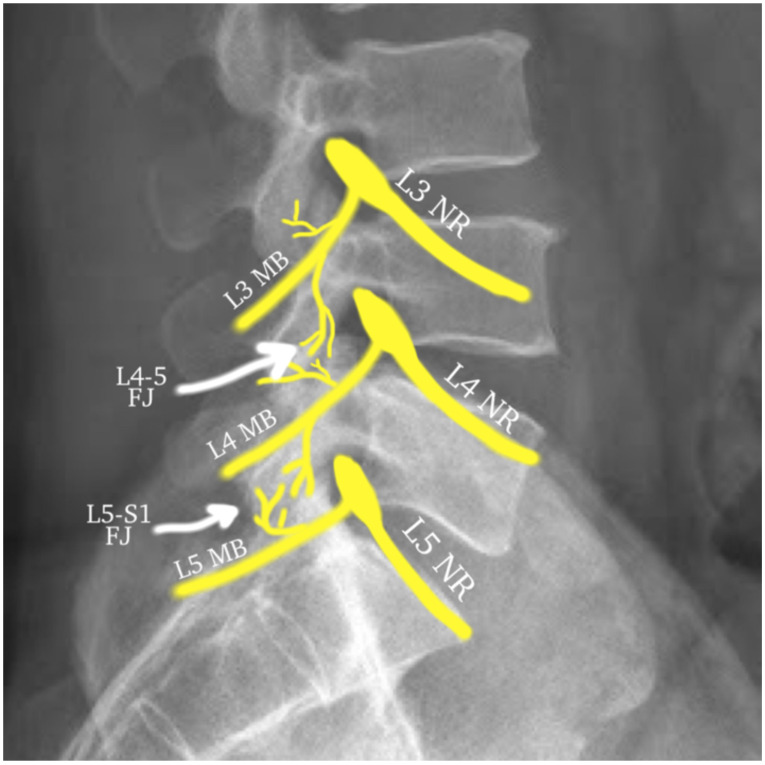
This diagram illustrates the dual innervation of the lumbar facet joints. The L4–L5 joint is receiving innervation from the L3 and L4 medial branches, while the L5-S1 joint is receiving innervation from the L4 medial branch and L5 dorsal ramus proper (prior to dividing to the L5 medial branch). Abbreviations: NR—nerve root; MB—medial branch; FJ—facet joint.

A common cause of posterior element pain stems from arthropathy of the lumbar facet (zygapophyseal) joints. The lumbar facet joints are true synovial joints with a hyaline cartilaginous surface, a fibrous capsule, and a distinct joint space that is capable of accommodating 1.0–1.5 mL of fluid. The facet joints form the posterolateral articulations, connecting the vertebral arch of one vertebra to that of the adjacent vertebra [136]. Each lumbar facet joint receives dual innervation by the medial branches of the posterior rami of the corresponding level and the level above and by the dorsal ramus proper at the L5 level (for instance, the L4–L5 facet joint is innervated by the L3 and L4 medial branches) (Figure 1) [137]. The lumbar facet joints have long been recognized as a significant cause of cLBP, as described as early as 1911 [138]. This was demonstrated further in 1963, when pain from the facet joints was reproduced when injected [139].

The spine moves as a three-joint complex composed of the intervertebral discs and the paired facet joints, which stabilize the spine and prevent excess movement in all planes. In young people, facet joints are strong and can support up to twice the person’s body weight [140]. As we age, the lumbar facet joints become more biplanar and transition from a largely coronal orientation to being more sagittal. Normal facet joints typically accept 3–25% of the axial load of the spine, and this number can increase significantly in the setting of degenerative discs [141]. Although specific injuries can occur to the facet joints, typically, pathology develops as a result of repetitive stresses as people age. Repetitive stress and subsequent inflammation can cause edematous stretching of the joint capsule and result in pain [142]. Similar to osteoarthritis in other joints throughout the body, the degenerative process can result in the formation of narrowed joint spaces, sclerosis and osteophytosis, and subchondral cyst formation. The derailment of normal physiologic facet joint functioning commonly results in facet joint syndrome being a significant contributor to cLBP, with prevalence increasing as the patient’s age increases [143].

As painful facet joint arthropathy can present similarly to osteoarthritis in other body locations, patients may complain of impaired mobility, low back stiffness (especially in the morning for less than 30 min), and mechanical pain that is worse with activity and repetitive range of motion of the lumbar spine [144]. Nonetheless, facetogenic low back pain can also arise as a result of inflammatory arthropathies, synovial impingement, synovitis, chronic infection, or synovial cysts, with variable presentations depending on facet joint pain etiology [145].

The prevalence of facet-generated cLBP varies widely, depending on the study methodology and patient populations, with prevalence reported as low as 4.8% and up to as much as 50% [146]. Lumbar facet joint syndrome is commonly described as bilateral or unilateral low back pain that can radiate or refer at times to the buttock, sides of the thighs, or groin, typically not extending beyond the knee, although, rarely, symptoms at the lower lumbar segments can refer to the lower leg [147]. It is important to note that facet joint arthropathy is a component of a generalized degenerative process of the spine and is rarely the sole contributor to patients’ pain (although at times, it can be predominant or contribute significantly). A prospective study of 206 patients suspected to have facetogenic pain underwent diagnostic blocks (either placebo-controlled comparative blocks or triple blocks), and only 15% of patients were determined to have purely facetogenic pain [148].

It has been concluded that radionuclide bone scintigraphy with single photon emission computed tomography, used to detect microcalcification due to increased osteoblastic activity, can aid in identifying potential painful generators in up to 86% of lumbar spine scans [149,150]. However, there have been inconclusive and conflicting results on the correlation between radiographing findings (on MRI, plain films, dynamic bending films, and CT) and facetogenic pain, with studies comparing imaging findings to placebo-controlled blocks or radiofrequency ablation. For that reason, the routine use of radiographic findings to diagnose facetogenic pain is not supported by the literature [151,152,153].

Therefore, a physical examination can aid in clinical suspicion when history and radiographic findings suggest potential painful facet joints. A physical exam may help delineate which patients may be indicated for potential diagnostic medial branch blocks as the gold standard for establishing diagnosis. When conceptualizing ways to reproduce facet joint pain, it is important to think about maneuvers that either distract or provide stress or compression to the suspected facet joints.

A cadaveric study by Khalsa et al. examined physiologic stresses to the facet joints with different movements. With lateral bending, the two most caudad facet joints (L4–L5 and L5-S1) showed the greatest magnitude of mechanical strain with contralateral bending, while L1–L3 joints demonstrated the greatest strain with ipsilateral bending. The movements with the largest intervertebral angulation were forward flexion for L4–L5, extension for L5-S1, ipsilateral lateral bending for L1–L2 and L3–L4, and contralateral lateral bending for L2–L3 [154]. Therefore, pain reproduced with these movements on a physical exam may increase suspicion of pain correlating to the expected joints. Importantly, Khalsa et al. also later confirmed in a follow-up study that the fusion of a lumbar segment resulted in increased strains noted at adjacent facet joints [155].

Kemp’s test is a common physical exam maneuver with the intention of compressing the lumbar facet joints. It is performed by placing the patient in lumbar extension, ipsilateral rotation, or obliquely oriented stress to reproduce ipsilateral low back pain. A systematic review evaluated five similar studies that compared the Kemp’s test to either single, dual, or comparative (two different anesthetics with varying duration) anesthetic blocks with at least 75% pain relief as the reference standard. The pooling of data concluded that the only diagnostic accuracy measure above 50% (56.8% and 59.9%) was its negative predictive value, indicating that the available literature suggests that the Kemp’s test has poor diagnostic value and limited utility [156].

When lumbar facet joint pain is suggested due to history, imaging, and physical exams, medial branch blocks remain the reference standard for diagnosis, as medial branch blocks are more predictive than intra-articular facet joint blocks. As therapeutic medial branch blocks and intra-articular facet joint injections have not shown convincing evidence for consistent long-term pain relief, the current treatment paradigm remains diagnostic medial branch blocks to confirm a diagnosis, with the treatment goal being radiofrequency denervation [157].

**Figure 2:**
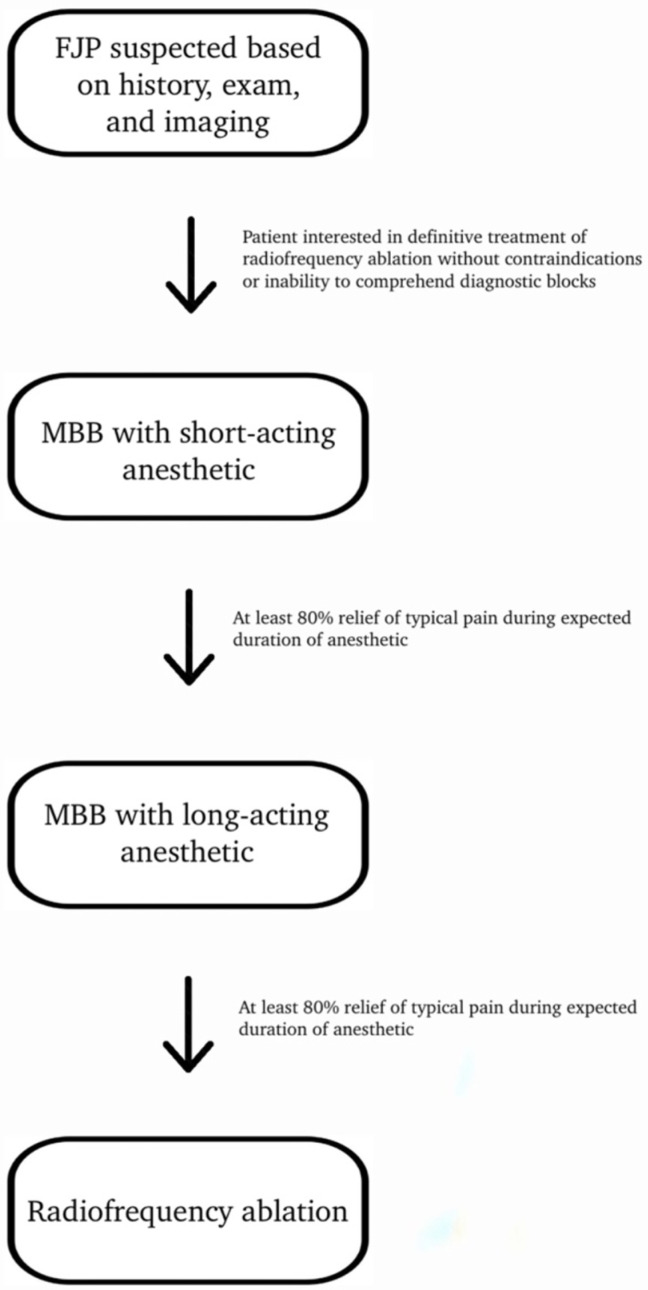
The algorithm depicting a typical treatment protocol for facet joint pain with the goal of radiofrequency ablation after the likelihood of diagnosis increased by two dual diagnostic blocks.

The most appropriate approach and interpretation of medial branch blocks for diagnosis and guiding treatment has been a topic of debate, and many issues are worth noting. Single diagnostic anesthetic blocks are not considered valid as they can have a high false-positive rate [158]. Additionally, lumbar medial branch blocks performed without controls have large false-positive rates (25–41%) [159,160]. More stringent diagnostic criteria may result in more false negatives but may improve outcomes for definitive treatment. Comparative anesthetic blocks with either long-acting or short-acting local anesthetic use on two separate occasions assessing pain relief duration corresponding to the anesthetic used have a sensitivity of 100% and a specificity of 65% [132]. The diagnostic confidence of these tests is complex, as sensitivity, specificity, and disease prevalence are all important factors that contribute to the validity of the test. As the disease prevalence increases, the false-positive rate will decrease, and the diagnostic confidence will increase. For instance, with the sensitivity and specificity listed above and a prevalence of 60%, the diagnostic confidence is 81%, and four out of five positive test results will be correct. If the prevalence drops to 15%, only one out of eight positive responses will truly be positive. As such, to avoid false-positive results, placebo-controlled blocks may be the best way to increase diagnostic confidence [161]. Unfortunately, insurers in the United States do not cover placebo-controlled blocks as the definitive procedure does not exceed the cost of blocks by a factor of 1.5, thus making placebo-controlled blocks not cost-effective [162]. As discussed below, radiofrequency ablation of the nerves innervating the facet joints is a common treatment for painful facet joints after diagnostic blocks have been performed. An example of a typical treatment paradigm for radiofrequency ablation can be seen in Figure 2.

Radiofrequency ablation is one of several interventional treatment options for facetogenic low back pain and is likely the most appropriate intervention for many patients, as randomized controlled trials (mentioned below) have shown limited evidence for other facet joint procedures. Although previous systematic reviews and meta-analyses have shown significant pain relief for 3–12 months, there is less evidence for functional improvement [163,164,165]. A Cochrane review from 2015 concluded that there is low-quality evidence for radiofrequency ablation for cLBP, but the available randomized controlled trials (RCTs) exhibit methodologic flaws and exhibit significant heterogeneity [166]. Similarly, the Mint RCTs concluded that radiofrequency ablation resulted in no improvement or no clinically important improvement in cLBP compared to a standardized exercise program alone [167]. Despite this, due to the reasons mentioned above regarding appropriate patient selection and the technically challenging nature of the procedure to place electrodes directly parallel to the medial branches to achieve sufficient ablation, there is a paucity of adequate trials to evaluate the effectiveness of this procedure.

Recently, endoscopic rhizotomy of the medial branches has gained interest as a treatment option, given the technical difficulty of radiofrequency ablation and in an effort to increase the duration of pain relief. A study of 55 patients diagnosed with a single medial branch block procedure with greater than 80% pain relief was non-randomized and received either conventional radiofrequency ablation or endoscopic rhizotomy. The median pain relief duration for the endoscopic rhizotomy group was twice that of the radiofrequency group, and 78.9% received greater than a 30-point Oswestry Disability Index decrease compared to 13.9% in the radiofrequency group. However, this study did not follow beyond 12 months; it had inadequate selection criteria; and it was not randomized or double blinded [168]. More well-designed studies evaluating the treatment of facetogenic low back pain need to be completed to determine the best treatment options.

Lastly, we will touch briefly on therapeutic intra-articular facet joint and medial branch injections. As the Centers for Medicare and Medicaid Services have decreased coverage for these treatments over the last few years, they are being performed sparingly in the United States at the time of this writing. In general, they are approved for patients who have had facet joint pain diagnosed with dual diagnostic blocks but are not candidates for radiofrequency ablation. Otherwise, intra-articular facet joint procedures can be utilized in the case of facet cysts, where rupture or aspiration can be attempted for radicular pain caused by the cyst impinging on exiting or traversing nerve roots. Several randomized controlled trials have failed to show a difference between saline, steroids, and anesthetics injected in facet joints [169,170,171]. Similarly, one small randomized controlled trial showed that intra-articular injection of steroids or saline did not result in a change in the need for eventual radiofrequency ablation [172]. The FACTS study, a double-blinded randomized controlled trial, found no significant difference for pain relief or functional outcome improvements between lumbar facet joint intra-articular injection with steroid or anesthetic or medial branch block with steroid, anesthetic, or saline for up to 6 months. The study also concluded that medial branch blocks for anesthetics or steroids did not perform better than saline [173]. Nonetheless, these procedures have been performed historically, and patients, anecdotally, have received significant relief, leaving them as a potential treatment option in the correct clinical context [157,174,175].

#### 7.2.2. Other Posterior Spinal Structures

The posterior ligamentous (interspinous and supraspinous ligaments) and osseous structures (spinous processes) of the spine are also potential sources of low back pain. Lumbar spine flexion is resisted by the ligamentum flavum and supraspinous and interspinous ligaments, based on biomechanical studies [176]. Ligamentopathia and sprains in the interspinous region are common causes of acute low back pain in athletes but can rarely result in intractable cLBP [177]. This pain is typically induced by lumbar flexion, and tenderness is elicited specifically in the interspinous region. 

Positional midline back pain with extension has at times been explained by Baastrup’s disease, or “Kissing spine disease”. This condition was first described by Christian Baastrup, a Danish radiologist, in 1933, and it is characterized on imaging by contacts between adjacent lumbar spinous processes causing radiographical findings such as sclerosis and enlargement and/or flattening of the appositional surfaces. Chronic repetitive stretching and weakening of the interspinous ligament can result in cyst formation ventrally near the posterior epidural space. Biomechanically, the interspinous and supraspinous ligaments that are degenerated in Baastrup’s disease normally contribute significantly to sagittal alignment [178].

This biomechanical role can be related to the radiographic findings seen in Baastrup’s disease and the different stages of the lumbar degenerative process with interspinous bursitis, interspinous fluid clefts, facet and dorsal epidural cysts, intervertebral disc space narrowing, ligamentous posterior spinal stenosis, and segmental instability with spondylolisthesis [179]. Therefore, Baastrup’s disease may be interpreted as a radiographic entity as a result of degenerative changes in the spine due to interspinous and supraspinous ligament mechanical derangements. In patients with “classical” Baastrup’s disease with midline localized positional low back pain in extension, it is important to consider more typical causes of pain such as lumbar facet arthropathy, degenerative spondylolisthesis, and lumbar spinal stenosis. 

With both Baastrup’s disease and ligamentous sprains, patients who fail to improve with conservative care of physical therapy and anti-inflammatory medications may be considered for interventional management. As this condition was originally described by relating the process to the spinous processes, treatment consisted of surgical removal of the spinous processes with inconsistent results. Only 11/64 patients who underwent this treatment reported pain relief [180]. Case studies have seen patients receive pain relief after fluoroscopically guided interspinous ligament steroid injections with aspiration attempted if cystic fluid is seen in the space on MRI [181,182]. If neurogenic claudication or radiculopathy are the most bothersome symptoms, patients may be considered for surgical stabilization or decompression [183].

## 8. Extra-Vertebral Column Pain

Structures further from the axial skeleton, or, more specifically, structures that are not directly a part of the vertebral column itself, are also common causes of cLBP. Although the etiology of low back pain can be attributed to numerous non-spinal causes (such as nephrolithiasis, endometriosis, tumors, etc.), we will focus this review primarily on the musculoskeletal causes of cLBP [184].

### 8.1. Sacroiliac Joint

Particularly, the sacroiliac joint is a common culprit of cLBP. The sacroiliac joint is an irregularly auricular-shaped joint surrounded by ligaments on both anterior and posterior surfaces. The sacroiliac joint complexes are continuations of the axial spine, continuing the kinetic chain through the lower extremities, with one of their major functions being to provide stability [185]. The sacroiliac joint is notorious for mimicking hip or spine pathology and also commonly arises as a result of hip dysfunction or spinal fusion. The sacroiliac joint has been estimated to contribute to 10–38% of LBP [186].

The sacroiliac joint can develop pain as a result of arthritis, enthesopathy, fractures, ligamentous injuries, and myofascial pain. In the correct clinical context, it may be necessary to evaluate patients for inflammatory spondyloarthropathies, as the sacroiliac joint is frequently affected in these conditions. Conditions that cause increased stress on the sacroiliac joint can also cause pain and include leg length discrepancy, abnormal gait, prolonged intense exercise, spinal deformity, pregnancy, spinal fusion, and hip pathology [187,188,189,190,191,192,193,194,195].

History and physical exams are vital to establishing a diagnosis of sacroiliac joint pain. Pain generated by the sacroiliac joint is typically located below the belt line, is aching in character without paresthesias, and can radiate into the groin or thigh and even beyond the knee. The sacroiliac joint can, at times, present with a radicular pattern of pain, which may be due to an insufficient capsular envelope with extravasation of inflammatory main mediators toward the dorsal sacral foramina, over the sacral ala to the L5 nerve root sheath, and ventrally toward the lumbosacral plexus [196]. Slipman et al. investigated referral patterns of sacroiliac joint pain and concluded that there are at least 18 different patterns of pain. Buttocks pain was the most common symptom (94%), followed by lower lumbar pain (72%), and groin pain (14%). Approximately 50% of patients had pain referring to the lower extremity, typically posterior and lateral thigh pain, but 14% of patients noted pain to the foot (with distal pain being seen more frequently in younger patients) [197]. Fortin et al. developed a pain referral map in asymptomatic volunteers after SIJ injection, with a hypesthetic area being identified as approximately 10 cm caudally and 3 cm laterally from the posterior superior iliac spine [198]. Therefore, when patients point to this area, it can be interpreted as a positive Fortin Finger Test [199].

Clinical history may reveal patients complaining of pain that is worse with prolonged static positions (commonly sitting but also standing or sleeping). Standing with more weight on one leg than the other may also intensify pain. Physical exam maneuvers aim to apply various stresses across the sacroiliac joint to provoke pain. Telli et al. investigated the validity and reliability of provocation tests (distraction test, compression test, thigh thrust test, sacral thrust test, and flexion abduction external rotation (FABER) test) in 156 patients, with the criterion being at least three positive provocative tests. Of these, when evaluated individually, FABER had the highest (91.4%) sensitivity, and the Gaenslen test had the lowest probability [200]. Schneider et al. evaluated FABER, thigh thrust, Gaenslen’s test, sacral distraction, lateral compression, and sacral thrust pre- and post-sacroiliac joint injection in 35 patients. The authors determined that no single exam maneuver had a positive likelihood ratio greater than 0.67, and the most sensitive tests were FABER and sacral thrust, while Gaenslen’s and thigh thrust were the least sensitive. They also determined that increasing the number of positive tests did not improve the diagnostic power, and the best likelihood ratio was 1.26 for greater than or equal to 5 positive tests, with no trend toward increasing the number of tests increasing the likelihood otherwise [201]. A systematic review and meta-analysis evaluated clusters of multiple pain provocation tests with single or double intra-articular anesthetic injections used as the reference standard in the included studies. The authors concluded that a positive cluster of provocative maneuvers can give the clinician 35% certainty of identifying sacroiliac joint pain. The clinical utility of a physical exam alone may be more pertinent to ruling out sacroiliac joint pain, as negative test maneuvers can conclude with 92% certainty that a negative test result is correct [202].

As there is inconsistent evidence for the utility of physical exam maneuvers, and as ultrasound and landmark-guided injections are not as accurate as injections performed with fluoroscopic guidance, fluoroscopically guided intra-articular diagnostic blocks remain the gold standard for the diagnosis of sacroiliac joint mediated pain [203]. Dual blocks with at least 70% pain relief provide Level II evidence for diagnostic accuracy, while single blocks with 75% pain relief provide Level III evidence [204,205]. For patients suspected to have sacroiliac joint pain and who have had inadequate improvement with conservative management (medications, therapy, osteopathic manipulation, sacroiliac joint belts, etc.), fluoroscopically guided injections with steroids can be both diagnostic and therapeutic. Ultrasound and landmark-guided injections can even be effective, as the posterior ligamentous complex can be a significant contributor to pain and can be effectively treated even in the absence of a confirmed intra-articular injection. Fluoroscopically guided steroid injections have shown significant pain relief and quality of life improvements up to 6 months or longer [206]. Injections of other substances, including dextrose for prolotherapy and platelet-rich plasma, have been used as potential therapeutic modalities, but limited data support their use, and there is insufficient evidence to support their use over steroid injections [207,208]. Radiofrequency ablation of the L4 medial branch, L5 dorsal ramus, and S1–S3 lateral branches, thought to provide sensation to the dorsal sacroiliac joint, has also been used as a treatment. Like the issues regarding facet joint radiofrequency ablation, the sacroiliac joint radiofrequency ablation procedure has had varying results, with studies showing limited evidence for its utility, and thus more rigorous studies need to be conducted to determine its effectiveness [167]. Surgical treatment is indicated in patients who have had a positive diagnostic response but have failed to obtain prolonged relief from conservative procedures or have contraindications to their use. Percutaneous arthrodesis may be a first-line surgical treatment, with open arthrodesis used in cases of revision or nonunion [186]. Minimally invasive sacroiliac joint fusion provides favorable responsiveness in quality of life metrics, pain scores, and overall postoperative outcomes in select patients and is probably more effective at reducing pain and opioid use when conservative management is inadequate, although adverse events appear to be higher through 6 months. Yet, prospective studies without industry influence need to be performed to determine the optimal treatment for patients with intractable sacroiliac joint pain [209,210].

### 8.2. Soft Tissue Structures

Finally, we will touch briefly on soft tissues contributing to cLBP. There are numerous myofascial, tendinous, and ligamentous structures within the low back to which we can attribute pain in patients. As a rule, we may be suspicious of this type of pain in patients who have biomechanical imbalances due to the pathology of adjacent areas, patients who have significant pain with palpation of these structures, or when forces are applied against these structures with stretching. Although muscular trigger points and tendinosis may be significant contributors to cLBP, we will focus on two specific etiologies: Maigne’s (or thoracolumbar junction) syndrome and iliolumbar ligament syndrome.

#### 8.2.1. Maigne’s Syndrome

Maigne’s syndrome is low back pain that is caused by pathology of the thoracolumbar dorsal ramus, entrapment of the superior cluneal nerve, subostal nerve, or iliohypogastric nerve at the thoracolumbar junction, and it can also present as pseudosciatica caused by the thoracolumbar lateral branch nerve [211,212]. Maigne’s syndrome may be underdiagnosed, as the pain is rarely felt at the thoracolumbar junction but may present in a location similar to other causes of lumbosacral cLBP. A prospective study of 834 consecutive patients in a surgical spine clinic determined the incidence of patients with Maigne syndrome to be 14% [213]. Additionally, it has been described that approximately 15–25% of pain in patients at the posterior iliac crest area results from injury or irritation of the posterior rami at the thoracolumbar junction due to fracture or degenerative changes, and this pain is often confused with lumbosacral region pain [214]. Although the exact mechanisms associated with Maigne’s syndrome remain unclear, it is agreed upon that the thoracolumbar junction is a transitional segment prone to rotational stresses. Disruption of transitional forces through the thoracolumbar junction can lead to segmental dysfunction, contributing to the syndrome [215].

The skin roll, also known as the pinch and roll test, should be used to aid in diagnosis. The skin is lifted, slightly pinched, and rolled, beginning at the flank and proceeding medially and inferiorly to the sacrum. If the patient responds sensitively to pain along the distribution of the above-mentioned nerves, the facet joints at the thoracolumbar junction should be palpated. Tenderness at the facet joints indicates a diagnosis of Maigne syndrome. The examiner also compares each side and notes where the skin is rolled more heavily [216,217].

Treatment involves the typical conservative management options for cLBP, including physical therapy and exercise programs, medications, spinal manipulative therapy, and acupuncture. If this fails, more invasive treatment options include ultrasound guided neural blocks, facet joint blocks, percutaneous rhizotomy, electrocoagulation, and surgical release of nerves [218].

#### 8.2.2. Iliolumbar Ligament Pain

The iliolumbar ligament is a ligamentous band that connects the transverse processes of the L4 and L5 vertebrae to the ilium on the same side and is an evolutionarily critical structure that allows for erect posture and bipedal movement [219,220].

Repetitive occupational microtrauma, acute strain, and/or poor posture have been postulated as possible etiologies for chronic iliolumbar syndrome [221]. As this structure inhibits sacral flexion, movements causing flexion strains in the sacrum may reproduce pain. Pain would be expected anatomically between the lower lumbar vertebrae and the ilium [222]. Likewise, tension is applied to the iliolumbar ligament via anteflexion and contralateral lateroflexion-rotation, which can reproduce painful symptomatology at the posteromedial iliac crest [223].

Suspicion for iliolumbar ligament pain can be increased when ultrasound reveals ligament thickening of 2 mm or greater at the posterosuperior iliac spine level, especially in the absence of X-ray or MRI evidence of other significant pathology (although the iliolumbar ligament may play a more important role in prohibiting anterior translation of the L5 vertebrae in the presence of spondylosis in this area). Anesthetic ultrasound-guided injections can aid in diagnosis and management [224].

#### 8.2.3. Spinal Segmental Instability and Muscular Dysfunction

Finally, altered back muscle structure and function are highly prevalent in patients with cLBP, particularly in the spinal extensor muscles: the lumbar multifidus and erector spinae [225]. The multifidus is the most medial lumbar spine muscle, with short, deep fibers that span two intervertebral segments and superficial muscles that span three to five vertebral segments [226]. The erector spinae is located laterally and consists of the lumbar and thoracic longissimus and iliocostalis muscle thoracic portions [226]. Extensive changes in back muscle structure and function are seen in patients with cLBP, such as a smaller cross-sectional area, fatty infiltration, and potentially increased type II muscle fiber types (seen in patients who were scheduled for spinal surgery) [227]. These changes can lead to impaired sensorimotor control and dysfunctional movements of the spine, which contribute to impaired stabilization and ultimately cLBP as they are instrumental in maintaining a pain-free range of motion, providing segmental stability in response to anticipated changes in posture, and protecting against sudden perturbations [228].

In regards to physical therapy for the above-mentioned nociceptive pain mechanisms mentioned throughout this review, specific exercises targeting the back muscles are probably more effective for these patients with cLBP of nociceptive origin [229]. Likewise, as interventions limited to back muscle exercises have been shown to improve pain and disability, this is likely an unideal treatment alone, and cLBP treatment must be multidimensional, taking into account the complex biopsychosocial environment surrounding chronic pain [230]. Back muscle dysfunction is not uniform, and treatment within this heterogenous group must depend on a thorough patient history and clinical examination. Similar to other neuromuscular disorders, impaired neuromuscular control can be seen as a consequence of derangement or injury of spinal structures, and activating these muscles can be difficult to achieve voluntarily [231]. Performing specific sensorimotor exercises at the initial stages of an exercise program may be useful to optimize specific muscle activation patterns and progress towards back muscle endurance and strength training [229].

## 9. Conclusions

Despite improvements in specialized spine care, history, diagnosis, and management remain challenging. A thorough knowledge of the differential diagnoses, their typical presentation, and imaging findings can aid in suspecting certain etiologies. When a specific condition is believed to be contributing to a patient’s symptoms, careful selection and execution of physical exam maneuvers, along with diagnostic procedures, can help confirm an appropriate diagnosis and guide treatment.
